# Gait abnormalities and non-motor symptoms predict abnormal dopaminergic imaging in presumed drug-induced Parkinsonism

**DOI:** 10.1038/s41531-022-00309-8

**Published:** 2022-04-28

**Authors:** Whitley W. Aamodt, Jacob G. Dubroff, Gang Cheng, Betty Taylor, Stephanie Wood, John E. Duda, James F. Morley

**Affiliations:** 1 Parkinson’s Disease Research, Education, and Clinical Center, Corporal Michael J. Crescenz VA Medical Center, 3900 Woodland Avenue, Philadelphia, PA 19104 USA; 2 Department of Neurology, University of Pennsylvania Perelman School of Medicine, 3W Gates, 3400 Spruce Street, Philadelphia, PA 19104 USA; 3 Department of Radiology, University of Pennsylvania Perelman School of Medicine, 1 Silverstein, 3400 Spruce Street, Philadelphia, PA 19104 USA; 4 Department of Radiology, Corporal Michael J. Crescenz Medical Center, Philadelphia, PA 19104 USA

**Keywords:** Parkinson's disease, Physical examination, Radionuclide imaging, Neurological manifestations

## Abstract

Drug-induced parkinsonism (DIP) can be clinically indistinguishable from degenerative parkinsonism, and bedside assessments are needed to differentiate between these conditions. We examined 34 U.S. Veterans with DIP using ^123^I-FP-CIT (DAT-SPECT) to identify underlying nigrostriatal degeneration. Participants were 94% male with mean age of 64.5 ± 7.1 years. DAT-SPECT was abnormal in 12/34 (35%). Comparing normal and abnormal imaging groups, there were no differences in age, sex, race/ethnicity, psychiatric diagnosis, motor severity, or RBD Screening Questionnaire scores. Those with underlying neurodegeneration reported significantly more non-motor symptoms (NMS), worse olfactory function on the University of Pennsylvania Smell Identification Test, and greater turning duration/steps on the instrumented Timed Up and Go. Area under the curve (AUC) combining poor olfaction and total NMS burden was 0.84 (CI 0.71–0.97), while AUC for turn steps was 0.91 (CI 0.81–1.00). Gait impairment, hyposmia, and NMS may be useful alone and in combination to identify DIP patients with underlying dopaminergic degeneration.

## Introduction

Parkinsonism is a clinical syndrome characterized by bradykinesia, rigidity, tremor, and postural instability. While idiopathic Parkinson’s disease (PD) is the most common cause of parkinsonism, a variety of neurodegenerative, structural, metabolic, paraneoplastic, or toxic conditions can affect the basal ganglia and nigrostriatal pathways and give rise to clinical symptoms^1^. Among these conditions, drug-induced parkinsonism (DIP) is the most common cause of non-degenerative parkinsonism and affects more than 15% of patients on long-term antipsychotic therapy^1,2^. DIP is most commonly associated with medications that affect the dopaminergic pathway, including mood stabilizers and dopamine receptor blocking agents (DRBAs) prescribed for psychiatric disorders and gastrointestinal complaints^1,3,4^.

DIP is often reversible within weeks to months of drug discontinuation. However, parkinsonism can also persist or worsen in some cases after drug withdrawal, suggesting that DIP may represent “unmasking” of subclinical nigrostriatal dysfunction consistent with incipient PD or another degenerative process such as dementia with Lewy bodies (DLB) or multiple system atrophy (MSA)^1,5,6^. Lewy pathology has also been described at autopsy in some cases of DIP^5^. Because DIP and PD or related disorders can be clinically indistinguishable, dopamine transporter imaging with ^123^I-FP-CIT (DAT-SPECT [single-photon emission computed tomography]) has emerged as the most reliable in vivo technique to distinguish between degenerative and non-degenerative forms of parkinsonism^7^. DAT-SPECT is used to assess the status of presynaptic nigrostriatal terminals, and abnormal results can reflect reduced dopamine transporter density and/or function^8^. In degenerative parkinsonism, abnormal DAT-SPECT often represents a reduction in presynaptic dopamine transporter density due to nigral cell loss^9^. In contrast, non-degenerative DIP is associated with pharmacologic blockade of postsynaptic nigrostriatal terminals, resulting in normal DAT-SPECT. Although pathologic diagnosis remains the gold standard for PD and related neurodegenerative disorders, DAT-SPECT is considered an indirect biomarker of degenerative parkinsonism and was incorporated into the diagnostic criteria for PD and DLB^10–12^. To this end, normal functional neuroimaging of the presynaptic dopaminergic system is considered an absolute exclusion criteria for the diagnosis of PD^11^. Unfortunately, functional imaging can be costly and is not available at all centers, making more accurate prediction of underlying neurodegeneration in DIP based on clinical motor and non-motor findings of particular importance for diagnosis, treatment, and prognostication.

Several studies have compared the motor and non-motor features of DIP and PD. In one small cohort, DIP was associated with upper extremity predominance, less tremor, and more symmetrical symptoms than PD^13^. In another case-control study comparing these conditions, PD patients were more likely to exhibit a postural instability/gait disorder phenotype, along with hyposmia, constipation, and impotence^14^. A follow-up study at our center also found that hyposmia among DIP patients predicted persistent parkinsonism after drug withdrawal and correlated with abnormal DAT-SPECT^15^, a result corroborated by a more recent study from Korea^16^. These findings suggest that olfactory dysfunction, a prodromal feature of Lewy body disorders, may predict underlying neurodegeneration. However, few studies have explored whether other motor and non-motor assessments correlate with abnormal imaging in DIP and whether group differences can translate into useful diagnostic testing for individual patients.

A recent retrospective chart review comparing DIP patients with normal and abnormal DAT-SPECT found that patients with abnormal imaging were more likely to have more than two cardinal motor manifestations of PD, while another study found that asymmetric motor symptoms, constipation, and urinary disturbances were more common in DIP patients with abnormal imaging^17,18^. However, DIP is often indistinguishable from degenerative parkinsonism in individual patients and data on the predictive accuracy of common clinical assessments are lacking. Our objectives in the current study were to (1) examine a cohort of United States Veterans with clinically diagnosed DIP using DAT-SPECT to identify those with or without underlying neurodegeneration, (2) characterize the pattern of nigrostriatal degeneration in those with abnormal DAT-SPECT, and (3) determine whether any objective clinical assessments, including quantitative gait analysis, either alone or in combination, can more accurately differentiate between degenerative and pharmacologic parkinsonism.

## Results

### Demographic and clinical characteristics

The mean age of participants was 64.5 ± 7.1 years, 32 (94%) were male, and 28 (82%) were white. The primary diagnoses for antidopaminergic drug use included bipolar disorder (32%), depression (23%), post-traumatic stress disorder (PTSD) (21%), schizophrenia (12%), schizoaffective disorder (3%), polysubstance abuse (6%), or epilepsy (3%). Among the 33 participants with a primary psychiatric disorder, 24% had associated psychotic features. In descending order, the most commonly prescribed medications were aripiprazole (35%), quetiapine (17%), risperidone (12%), olanzapine (6%), valproic acid (6%), haloperidol (3%), lithium (3%), lurasidone (3%), or multiple agents (15%).

Overall, 12 (35%) DAT-SPECT scans were read as abnormal, consistent with underlying neurodegeneration. When comparing participants with or without underlying dopaminergic deficiency, there were no differences in age, sex, race/ethnicity, underlying diagnosis, or the presence of psychotic features. In addition, the proportion of participants taking antidepressants that could influence DAT-SPECT results, including selective serotonin reuptake inhibitors (SSRIs), were similar between groups. However, the intensity of antipsychotic treatment, represented by chlorpromazine equivalents, was significantly lower in participants with abnormal imaging (152.5 ± 91.6 mg vs. 308.3 ± 164.8 mg, *p* = 0.001). Demographic and clinical characteristics are summarized in Table 1.

**Table 1:** Demographic and clinical characteristics of DIP patients with normal and abnormal DAT‐SPECT.

	Normal DAT‐SPECT *n* = 22/34 (65%)	Abnormal DAT‐SPECT *n* = 12/34 (35%)	*p* value
Age, years	64.1 (6.8)	65.3 (8.1)	0.671
Sex, *n* (%) male	21 (95%)	11 (92%)	0.654
Race/ethnicity, *n* (%) white	19 (86%)	9 (75%)	0.406
Diagnosis, *n* (%)			
Bipolar disorder	8 (36%)	3 (25%)	0.151
Depression	4 (18%)	4 (33%)	
Post-traumatic stress disorder	5 (23%)	2 (17%)	
Schizophrenia	4 (18%)	0	
Schizoaffective disorder	0	1 (8%)	
Other psychoses	0	2 (17%)	
Epilepsy	1 (5%)	0	
Psychosis, *n* (%)	6 (27%)	2 (17%)	0.681
Dose, CPZ equivalents, mg	308.3 (164.8)	152.5 (91.6)	**0.001**
DAT interfering drug, %	11 (50%)	8 (67%)	0.350
UPDRS-Part III	14.5 (7.0)	19.3 (6.0)	0.069
NMSQ total	11.4 (6.8)	16.5 (5.7)	**0.033**
RBDSQ total	6.1 (3.2)	7.8 (3.6)	0.175
UPSIT percentile	46.2 (23.4)	27.6 (22.1)	**0.031**

Bold values indicates statistical significant *p* < 0.05.

Group comparisons were assessed using chi‐square analysis and Fisher’s exact test for categorical variables, independent‐sample *t*-tests for normally distributed continuous variables, and Wilcoxon rank-sum for non-normally distributed continuous variables. Chlorpromazine equivalents were calculated for 31 of 34 participants.

Data are mean (standard deviation) unless otherwise noted.

### Pattern of DAT-SPECT uptake

Age-adjusted *Z*-scores for DAT-SPECT uptake were lower for abnormal scans in all regions of the basal ganglia (striatum *p* = 0.001, caudate *p* = 0.003, anterior putamen *p* < 0.001, posterior putamen *p* < 0.001). Regional mean differences between normal and abnormal scans were most pronounced in the posterior putamen. In addition, differences were greater when comparing the putamen to caudate and posterior putamen to anterior putamen, though these differences did not reach statistical significance. Mean differences and standard errors are summarized in Table 2.

**Table 2:** Regional differences in DAT-SPECT uptake between those with normal and abnormal imaging.

Region	Normal DAT-SPECT (*n* = 22)	Abnormal DAT-SPECT (*n* = 11)	Mean difference (normal-abnormal)	SE difference	*p* value
Striatum	0.846	−1.530	2.376	0.836	**0.0010**
Caudate	0.725	−1.425	2.151	0.864	**0.0033**
Anterior Putamen	0.532	−1.724	2.256	0.843	**0.0007**
Posterior Putamen	1.133	−1.364	2.497	0.744	**0.0003**

Bold values indicates statistical significant *p* < 0.05.

Group comparisons were assessed using Wilcoxon rank-sum for non-parametric data.

### Clinical features that distinguish DIP from underlying dopaminergic deficiency

With regard to motor and non-motor symptoms (NMS), total Unified Parkinson’s Disease Rating Scale Part III (UPDRS-III) and RBD Screening Questionnaire (RBDSQ) scores did not differ between participants with normal and abnormal DAT-SPECT. However, those with evidence of underlying neurodegeneration had lower University of Pennsylvania Smell Identification Test (UPSIT) age- and sex-adjusted percentile scores (*p* = 0.031) and a higher total burden of NMS represented by NMSQ scores (*p* = 0.033), which are summarized in Table 1. When assessing individual NMS on the NMSQ, participants with abnormal imaging were more likely to report incomplete bowel emptying (*p* = 0.003), depression (*p* = 0.027), and dream enactment (*p* = 0.026). Itemized NMSQ responses are listed in Table 3.

**Table 3:** Itemized NMSQ responses in DIP patients with normal and abnormal DAT‐SPECT.

	Normal DAT‐SPECT (*n* = 22)	Abnormal DAT‐SPECT (*n* = 12)	*p* value
NMSQ total	11.4 (6.8)	16.5 (5.7)	**0.033**
Q1. Dribbling of saliva during the daytime	8 (36%)	6 (50%)	0.440
Q2. Loss or change in ability to taste or smell	4 (18%)	5 (42%)	0.224
Q3. Difficulty swallowing food or drink or problems with choking	6 (27%)	3 (25%)	1.000
Q4. Vomiting or feelings of sickness	5 (23%)	5 (42%)	0.247
Q5. Constipation (less than 3 bowel movements a week) or having to strain to pass a stool	5 (23%)	6 (50%)	0.104
Q6. Bowel incontinence	4 (18%)	6 (50%)	0.112
Q7. Feeling that your bowel emptying is incomplete after having been to the toilet	5 (23%)	9 (75%)	**0.003**
Q8. A sense of urgency to pass urine makes you rush to the toilet	12 (55%)	10 (83%)	0.093
Q9. Getting up regularly at night to pass urine	10 (45%)	9 (75%)	0.097
Q10. Unexplained pains (not due to known conditions such as arthritis)	10 (45%)	5 (42%)	0.832
Q11. Unexplained change in weight	3 (14%)	5 (42%)	0.098
Q12. Problems remembering things that have happened recently or forgetting to do things	17 (77%)	10 (83%)	0.676
Q13. Loss of interest in what is happening around you or doing things	15 (68%)	7 (58%)	0.566
Q14. seeing or hearing things that you know or are told are not there	6 (27%)	5 (42%)	0.391
Q15. Difficulty concentrating or staying focused	14 (64%)	10 (83%)	0.228
Q16. Feeling sad, ‘low’ or ‘blue’	12 (55%)	11 (92%)	**0.027**
Q17. Feeling anxious, frightened or panicky	15 (68%)	9 (75%)	0.677
Q18. Feeling less interested in sex or more interested in sex	11 (50%)	5 (42%)	0.642
Q19. Finding it difficult to have sex when you try	12 (55%)	8 (67%)	0.493
Q20. Feeling light headed, dizzy or weak standing from sitting or lying	10 (45%)	8 (67%)	0.236
Q21. Falling	6 (27%)	6 (50%)	0.185
Q22. Finding it difficult to stay awake during activities such as working, driving or eating	5 (23%)	3 (25%)	1.000
Q23. Difficulty getting to sleep at night or staying asleep at night	10 (45%)	7 (58%)	0.473
Q24. Intense, vivid dreams or frightening dreams	13 (14%)	9 (75%)	0.354
Q25. Talking or moving about in your sleep as if you are ‘acting’ out dreams	6 (27%)	8 (67%)	**0.026**
Q26. Unpleasant sensations in your legs at night or while resting, and a feeling that you need to move	11 (50%)	9/12 (75%)	0.157
Q27. Leg swelling	6 (27%)	3 (25%)	1.000
Q28. Excessive sweating	5 (23%)	5 (42%)	0.247
Q29. Double vision	2 (9%)	4 (33%)	0.154
Q30. Believing things are happening to you that other people say are not true	2 (9%)	2 (17%)	0.602

Bold values indicates statistical significant *p* < 0.05.

Group comparisons were assessed using chi‐square analysis or Fisher’s exact test based on sample size.

Multiple parameters of gait analysis using the instrumented Timed Up and Go (iTUG) differed between groups and are summarized in Table. In particular, many components of the turning phase, including turn steps (*p* < 0.001), turn duration (*p* = 0.011), and turn-to-sit duration (*p* = 0.039), were greater among DIP participants with underlying dopaminergic deficiency.

**Table 4:** Instrumented Timed Up and Go Test (iTUG) performance of DIP patients with normal and abnormal DAT‐SPECT.

Gait metric	Normal DAT‐SPECT (*n* = 19)	Abnormal DAT‐SPECT (*n* = 9)	*p* value
Total duration, sec	22.67 (4.43)	26.30 (4.07)	**0.048**
Sit to stand duration, sec	2.36 (0.41)	2.43 (0.46)	0.670
Stride length, % stature^a^	74.72 (10.36)	68.51 (8.40)	**0.046**
Arm swing range of motion, degrees	21.81 (7.86)	20.43 (4.54)	0.630
Cadence, steps/min	97.26 (9.09)	98.43 (11.67)	0.774
Turn steps, #	5.29 (0.98)	6.86 (0.82)	**<0.001**
Turn duration, sec	2.97 (0.74)	3.77 (0.65)	**0.011**
Turn-to-sit duration, sec	5.02 (1.23)	6.01 (0.82)	**0.039**

Bold values indicates statistical significant *p* < 0.05.

Group comparisons were assessed using independent‐sample *t*-tests for normally distributed continuous variables and Wilcoxon rank-sum for non-normally distributed continuous variables, denoted by^a^. Gait data were available for 28 of 34 participants.

Data are mean (standard deviation) unless otherwise noted.

### Predictive capacity of clinical features alone or in combination

**Fig. 1:**
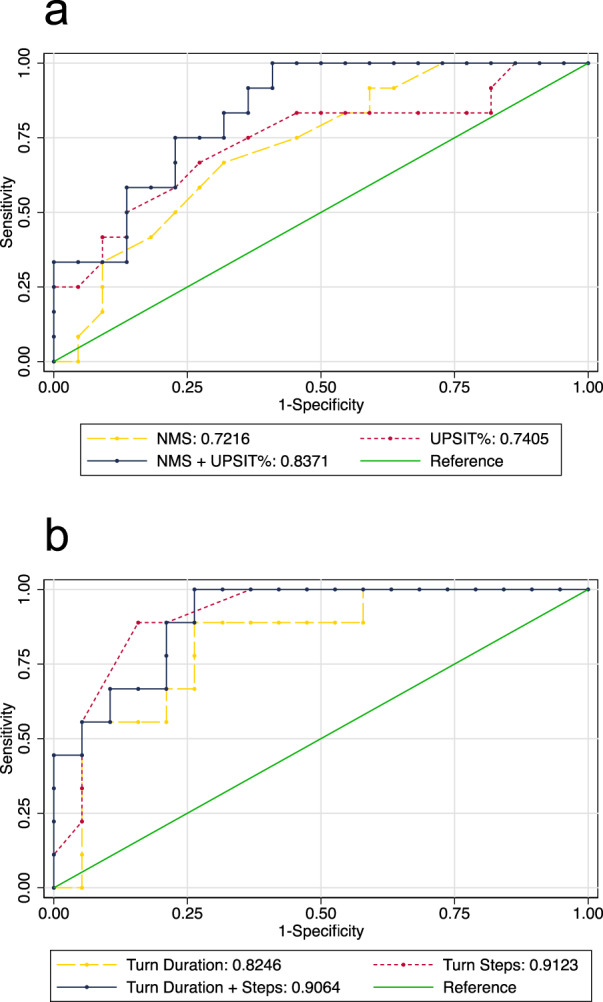
ROC curves. Receiver operating characteristic (ROC) curves for clinical predictors of abnormal DAT-SPECT in DIP patients. Non-motor ROC curves include total score on the Non-Motor Symptoms Questionnaire (NMS), age- and sex-adjusted percentile score on the University of Pennsylvania Smell Identification Test (UPSIT%), and a combined measure of NMS total and UPSIT percentile scores (**a**). Motor ROC curves include turn duration, turn steps, and a combined measure of turn duration and steps using the instrumented Timed Up and Go (iTUG) (**b**).

To determine whether clinical assessments can accurately differentiate between degenerative or pharmacologic parkinsonism, receiver operating characteristic (ROC) curve analysis was performed using the measures that differed between participants with and without abnormal imaging: olfactory performance, total NMSQ score, turn steps, and turn duration measured during the iTUG. ROC analysis was not performed for individual items on the NMSQ to limit multiplicity. In ascending order, the individual areas under the curve (AUCs) were 0.72 (CI 0.55–0.90) for NMSQ total score, 0.74 (CI 0.55–0.93) for UPSIT age- and sex-adjusted percentile score, 0.82 (CI 0.66–0.99) for turning duration in seconds, and 0.91 (CI 0.81–1.00) for number of turn steps. Combined AUCs were 0.84 (CI 0.71–0.97) for NMSQ total score and UPSIT age- and sex-adjusted percentile score, 0.91 (CI 0.80–1.00) for turning duration and turn steps, and 0.92 (CI 0.83–1.00) for all variables. Non-motor and motor ROC curves are shown in Fig. 1a, b, respectively.

Next, given the greatest accuracy of turn steps in predicting dopaminergic deficit, the optimal cut-point was calculated using Youden’s Index. While the optimal cut-point was 6.17 steps on the iTUG with 89% sensitivity and 84% specificity, a cut-point of 6 steps or greater is more easily calculated in routine clinical settings and differentiated between pharmacologic and degenerative parkinsonism with 89% sensitivity and 79% specificity.

## Discussion

DIP is the second most common cause of parkinsonism after idiopathic PD and is associated with significant psychiatric and neurologic morbidity. Emerging parkinsonism in patients treated with DRBAs may represent one of at least two phenomena: (1) blockade of postsynaptic dopamine receptors that resolves following removal of an offending agent consistent with uncomplicated DIP, or (2) “unmasking” of subclinical presynaptic nigrostriatal degeneration that persists or worsens independent of drug withdrawal suggestive of underlying neurodegeneration^7^. DIP and “unmasking” can be clinically indistinguishable but have dramatically different prognostic and management implications; thus, identifying biomarkers or clinical features that distinguish between these entities represents a significant unmet need.

DAT-SPECT has emerged as a potentially valuable modality to differentiate between pharmacologic and degenerative parkinsonism and several studies have demonstrated dopaminergic denervation in a substantial fraction of cases (~20–40%)^15,17,19–22^. However, DAT-SPECT is not available in many centers and may not be desirable in all clinical scenarios, prompting the need for other assessments that can accurately distinguish between these conditions. We identified several clinical features that differed between DIP participants with and without underlying dopaminergic denervation. Notably, the intensity of antidopaminergic treatment, represented by chlorpromazine equivalents, was significantly lower in participants with abnormal imaging, signifying less dopaminergic blockade in DIP patients with underlying denervation. This finding may reflect greater sensitivity to DRBAs in patients with subclinical nigrostriatal degeneration, as psychiatric diagnosis was not associated with DAT-SPECT result. In such cases, dopamine blockers may act as a “stress test” for the nigrostriatal pathway by unmasking otherwise subclinical abnormalities that serve as an important clue when parkinsonism emerges at lower intensity antidopaminergic treatment^15^.

If some cases of DIP represent unmasking of incipient PD and related disorders by DRBAs, one might expect features of prodromal PD to be over-represented in DIP patients with abnormal DAT-SPECT. Indeed, we and others have observed that hyposmia, a frequent prodromal feature of PD, is more common in participants with dopaminergic degeneration^14,15,23,24^. In one small study, 14 of 15 subjects with DIP had normal olfaction, but one subject with anosmia had evidence of cardiac sympathetic denervation, another early prodromal feature of PD^23^. In another small cohort that measured olfaction using the “Sniffin’ Sticks” test, DIP patients with abnormal SPECT had olfactory impairment that correlated with reduced putaminal uptake^24^. Interestingly, only 42% of our study participants with abnormal DAT-SPECT self-reported hyposmia or dysgeusia on the NMSQ, similar to the relative agnosia of sensory dysfunction in PD (Table 3)^25^. This finding supports the use of objective measures of olfaction to detect olfactory abnormalities associated with underlying dopaminergic degeneration.

In addition to olfactory impairment, several self-reported NMS often observed as prodromal features of PD and related disorders were more common in those with abnormal imaging, including incomplete bowel emptying, depression, and dream enactment (Table 3). Another study using the Non-Motor Symptoms Scale found that hyposmia, urinary symptoms, and excessive daytime sleepiness were greater in patients with PD compared to DIP^26^. Although PD is strongly associated with constipation, depression, and REM behavior disorder and likely explains our study findings, patients with underlying psychiatric diagnoses may also experience mood symptoms and abnormal sleep behaviors. For example, untreated psychiatric symptoms in our study cohort may have influenced self-reported depression on the NMSQ; however, antidepressant use did not differ between groups. In addition, although participants with abnormal DAT-SPECT were more likely to report dream enactment on the NMSQ, RBDSQ score was not sensitive to differences in DAT-SPECT in the current study. These findings may be explained by the high prevalence of sleep disorders in the general Veteran population, including PTSD-associated parasomnias and nightmares. Given some variability in self-reported NMS, aggregate non-motor scores may be more reliable in predicting underlying nigrostriatal degeneration.

Although there were no differences in overall motor function, DIP patients with underlying neurodegeneration demonstrated changes in quantitative gait function and performed worse on turning measures during the iTUG. In particular, patients with abnormal imaging took more steps when turning and had longer turn times based on wearable sensor data (Table 4). These changes may foreshadow “en bloc” turning seen in patients with PD and related disorders. Similarly, we previously described higher gait scores in patients with persistent parkinsonism after drug withdrawal^14^, suggesting that gait dysfunction may help differentiate between pharmacologic and degenerative parkinsonism. In addition, one prior study comparing DIP and PD patients on the iTUG test using a smartphone-based motion capture system found that DIP and PD patients differed from healthy controls on several gait metrics^27^. Although turning data were not reported, this study supports the use of wearable sensor-based technology for assessing gait and balance. In the current investigation, the number of turn steps was associated with an AUC >0.9, suggesting that this clinical feature alone may be useful on an *individual* basis to clarify the underlying diagnosis in DIP. Turn steps could potentially be calculated with or without wearable sensors, though gait metrics should be validated in independent cohorts^27^.

Our study has several limitations. First, the Veteran cohort was largely male, while DIP is more common in women, potentially limiting generalizability. In addition, more than half of the patients studied were taking medications (predominantly SSRIs) that could result in up to 10–15% alterations in DAT binding^19,28^. While this interaction does not typically impact routine DAT-SPECT interpretation, it may have impacted our semi-quantitative analyses. The proportion of participants on potentially interfering medications did not differ between groups, but this observation could be attributed to lack of power. Third, abnormal DAT-SPECT is not diagnostic for PD and our cohort has not been followed longitudinally to establish whether participants ultimately met clinical criteria for PD or another atypical parkinsonian syndrome. Finally, we present findings from a single-center study and results should be validated in larger cohorts.

Prominent gait symptoms and a higher burden of NMS may be useful alone and in combination to identify DIP patients with underlying dopaminergic degeneration. Because patients with DIP may also represent an “at-risk” cohort for the development of PD and related disorders, these clinical assessments could be used for longitudinal screening or to risk-stratify and guide further workup, including DAT-SPECT or empiric therapies. Given the apparent high accuracy of quantitative gait measures in predicting abnormal DAT-SPECT, future studies should validate the diagnostic value of these modalities and determine whether simply counting the number of steps required to turn during a clinical encounter might have similar diagnostic value. Longitudinal studies are also needed to explore outcomes in DIP patients with abnormal imaging, along with the emergence of other motor and non-motor features that may aid in diagnosis and prognostication.

## Methods

### Participants

We prospectively enrolled 34 participants at the Corporal Michael J. Crescenz VA Medical Center and Parkinson’s Disease Research, Education, and Clinical Center with a clinical diagnosis of DIP. Inclusion criteria were: (1) age 45–89 years; (2) development of clinical parkinsonism after the institution of pharmacologic therapy having known dopamine receptor blocking activity (antipsychotics, metoclopramide) or known association with DIP (lithium, valproic acid). Exclusion criteria were: (1) known diagnosis of PD, atypical parkinsonian syndrome (e.g., DLB, MSA, progressive supranuclear palsy, corticobasal degeneration) or other neurodegenerative condition; (2) known olfactory deficit due to surgery, trauma, infection or other etiology; (3) contraindication to DAT-SPECT. Participants taking medications with a major effect on DAT-SPECT (e.g., methylphenidate or other stimulants, benztropine, bupropion) were not enrolled. Participants taking medications with minor theoretical effects (<10–15%, e.g., SSRI antidepressants) were enrolled^28^. Study procedures were approved by the Corporal Michael J. Crescenz VA Medical Center institutional review board, and written informed consent was obtained from all participants.

### Data acquisition

#### Clinical and demographic assessments

A standardized template was used to extract the following variables from the electronic medical record: demographics, psychiatric diagnosis and treatment, offending agent and dose (normalized using chlorpromazine equivalents, where possible), and interfering medications. Motor function was assessed using the UPDRS-III^29^ and gait data were collected using Opal sensors during the iTUG and analyzed with APDM’s Mobility Lab^TM^ (APDM Inc., Portland, OR, USA). NMS were assessed using the NMSQ and RBDSQ, while olfactory function was assessed using the 40-item UPSIT. UPSIT raw scores were converted to age- and sex-specific percentiles based on normative data^30^.

#### Dopamine transporter SPECT

Patients received oral Lugol’s solution ~1 h before the study and were scanned on a dual-headed Symbia gamma camera (Siemens USA, Washington, D.C.) ~3 h after injection of 3–5 mCi of ^123^I-ioflupane. Images were acquired over 360° using low energy high-resolution collimators, a 128 × 128 matrix, 64 views per camera head (128 views total). The camera heads were kept as close as possible to the patient’s head (11–15 cm). Each projection was acquired for 30 s with a 159 keV ± 10% window. SPECT images were reconstructed via filtered back projection using Chang’s attenuation correction (attenuation coefficient µ of 0.11 cm^– 1^). Scans were evaluated by a nuclear medicine physician (JGD, GC) and read as normal or abnormal without knowledge of clinical status. Semi-quantitative analysis of attenuation-corrected DAT-SPECT uptake in four regions of the basal ganglia (striatum, caudate, anterior and posterior putamen) was acquired using MIMNeuro^®^ and reported as age-adjusted *Z*-scores.

### Statistical analysis

After identifying participants with normal and abnormal imaging, baseline demographic and clinical characteristics, UPDRS-III total score, quantitative gait data, NMSQ total score, RBDSQ total score, UPSIT percentile score, and age-adjusted *Z*-scores for DAT-SPECT uptake were compared using chi‐square analysis or Fisher’s exact test for categorical variables, independent‐sample *t*-tests for normally distributed continuous variables, and Wilcoxon rank-sum for non-normally distributed continuous variables. Based on significant findings, ROC curve analysis was performed to calculate the AUC and subsequent accuracy of motor symptoms, NMS, or a combination in predicting abnormal DAT-SPECT and corresponding nigrostriatal degeneration. All statistical tests were two-sided and significance was set at *p* < 0.05 using Stata (v16; StataCorp, College Station, TX).

### Reporting summary

Further information on research design is available in the Nature Research Reporting Summary linked to this article.

## Supplementary information

Reporting Summary

## Data Availability

The datasets generated and/or analyzed during the current study are available from the corresponding author on reasonable request.
