# Supplementary material for: Gait abnormalities and non-motor symptoms predict abnormal dopaminergic imaging in presumed drug-induced Parkinsonism
Source: NPJ Parkinsons Dis. 2022 Apr 28;8:53. doi: 10.1038/s41531-022-00309-8 (PMC9051164; doi:10.1038/s41531-022-00309-8)
Supplement: Supplementary file 1 — Reporting Summary [file 41531_2022_309_MOESM1_ESM.pdf]

nature portfolio

Corresponding author(s): James Morley, MD, PhD

Last updated by author(s): Dec 11, 2021

# Reporting Summary

Nature Portfolio wishes to improve the reproducibility of the work that we publish. This form provides structure for consistency and transparency in reporting. For further information on Nature Portfolio policies, see our Editorial Policies and the Editorial Policy Checklist.

## Statistics

For all statistical analyses, confirm that the following items are present in the figure legend, table legend, main text, or Methods section.

| n/a | Confirmed | |
|---|---|---|
| ☐ | ☒ | The exact sample size (*n*) for each experimental group/condition, given as a discrete number and unit of measurement |
| ☐ | ☒ | A statement on whether measurements were taken from distinct samples or whether the same sample was measured repeatedly |
| ☐ | ☒ | The statistical test(s) used AND whether they are one- or two-sided<br>*Only common tests should be described solely by name; describe more complex techniques in the Methods section.* |
| ☐ | ☒ | A description of all covariates tested |
| ☐ | ☒ | A description of any assumptions or corrections, such as tests of normality and adjustment for multiple comparisons |
| ☐ | ☒ | A full description of the statistical parameters including central tendency (e.g. means) or other basic estimates (e.g. regression coefficient) AND variation (e.g. standard deviation) or associated estimates of uncertainty (e.g. confidence intervals) |
| ☐ | ☒ | For null hypothesis testing, the test statistic (e.g. *F*, *t*, *r*) with confidence intervals, effect sizes, degrees of freedom and *P* value noted<br>*Give P values as exact values whenever suitable.* |
| ☒ | ☐ | For Bayesian analysis, information on the choice of priors and Markov chain Monte Carlo settings |
| ☒ | ☐ | For hierarchical and complex designs, identification of the appropriate level for tests and full reporting of outcomes |
| ☒ | ☐ | Estimates of effect sizes (e.g. Cohen's *d*, Pearson's *r*), indicating how they were calculated |

*Our web collection on statistics for biologists contains articles on many of the points above.*

## Software and code

Policy information about availability of computer code

| | |
|---|---|
| Data collection | Gait data were collected using Opal sensors during the instrumented Timed Up and Go and analyzed with APDM's Mobility LabTM (APDM Inc., Portland, OR, USA). |
| Data analysis | All statistical tests were two-sided and significance was set at p<0.05 using Stata (v16; StataCorp, College Station, TX). |

For manuscripts utilizing custom algorithms or software that are central to the research but not yet described in published literature, software must be made available to editors and reviewers. We strongly encourage code deposition in a community repository (e.g. GitHub). See the Nature Portfolio guidelines for submitting code & software for further information.

## Data

Policy information about availability of data

All manuscripts must include a data availability statement. This statement should provide the following information, where applicable:

- Accession codes, unique identifiers, or web links for publicly available datasets
- A description of any restrictions on data availability
- For clinical datasets or third party data, please ensure that the statement adheres to our policy

The datasets generated and/or analyzed during the current study are available from the corresponding author on reasonable request.

# Field-specific reporting

Please select the one below that is the best fit for your research. If you are not sure, read the appropriate sections before making your selection.

☒ Life sciences ☐ Behavioural & social sciences ☐ Ecological, evolutionary & environmental sciences

For a reference copy of the document with all sections, see nature.com/documents/nr-reporting-summary-flat.pdf

# Life sciences study design

All studies must disclose on these points even when the disclosure is negative.

| | |
|---|---|
| Sample size | 34 patients with presumed drug-induced parkinsonism who underwent DAT-SPECT were included in the study and analysis. The minimum sample size to detect a mean difference of 1.2 using a two-sample mean test with 80% power and an alpha level of 0.05 is 24. Thus, our sample size was deemed sufficient to detect differences in demographic and clinical data. |
| Data exclusions | No data were excluded from the analyses. |
| Replication | This study is a single-center study and results should be validated in independent cohorts. This statement was included in the text. |
| Randomization | No randomization was performed, as this study was a cohort study. |
| Blinding | All patients undergoing imaging (DAT-SPECT) were clinically diagnosed with drug-induced parkinsonism (DIP). However, when interpreting imaging result, nuclear medicine physicians were blinded to the patient's clinical status. |

# Reporting for specific materials, systems and methods

We require information from authors about some types of materials, experimental systems and methods used in many studies. Here, indicate whether each material, system or method listed is relevant to your study. If you are not sure if a list item applies to your research, read the appropriate section before selecting a response.

## Materials & experimental systems

| n/a | Involved in the study | |
|---|---|---|
| ☒ | ☐ | Antibodies |
| ☒ | ☐ | Eukaryotic cell lines |
| ☒ | ☐ | Palaeontology and archaeology |
| ☒ | ☐ | Animals and other organisms |
| ☐ | ☒ | Human research participants |
| ☐ | ☒ | Clinical data |
| ☒ | ☐ | Dual use research of concern |

## Methods

| n/a | Involved in the study | |
|---|---|---|
| ☒ | ☐ | ChIP-seq |
| ☒ | ☐ | Flow cytometry |
| ☒ | ☐ | MRI-based neuroimaging |

## Human research participants

Policy information about studies involving human research participants

| | |
|---|---|
| Population characteristics | The mean age of our 34 participants was 64.5±7.1 years, 32 (94%) were male, and 28 (82%) were white. The primary diagnoses for anti-dopaminergic drug use included bipolar disorder (32%), depression (23%), post-traumatic stress disorder (PTSD) (21%), schizophrenia (12%), schizoaffective disorder (3%), polysubstance abuse (6%), or epilepsy (3%). Among the 33 participants with a primary psychiatric disorder, 24% had associated psychotic features. In descending order, the most commonly prescribed medications were aripiprazole (35%), quetiapine (17%), risperidone (12%), olanzapine (6%), valproic acid (6%), haloperidol (3%), lithium (3%), lurasidone (3%), or multiple agents (15%). Overall, 12 (35%) DAT-SPECT scans were read as abnormal, consistent with underlying neurodegeneration. When comparing participants with or without underlying dopaminergic deficiency, there were no differences in age, sex, race/ethnicity, underlying diagnosis, or the presence of psychotic features. In addition, the proportion of participants taking antidepressants that could influence DAT-SPECT results, including selective serotonin reuptake inhibitors (SSRIs), were similar between groups. |
| Recruitment | We prospectively enrolled 34 participants at the Corporal Michael J. Crescenz VA Medical Center (VAMC) and Parkinson's Disease Research, Education, and Clinical Center (PADRECC) with a clinical diagnosis of drug-induced parkinsonism during clinical visits. All patients were U.S. Veterans and approached at the Philadelphia VAMC. In addition, the majority of patients were male, which may limit the generalizability of results to females and non-VA populations. |
| Ethics oversight | Study procedures were approved by the local Philadelphia VAMC institutional review board, and written informed consent was obtained from all participants. |

Note that full information on the approval of the study protocol must also be provided in the manuscript.

# Clinical data

Policy information about clinical studies

All manuscripts should comply with the ICMJE guidelines for publication of clinical research and a completed CONSORT checklist must be included with all submissions.

| | |
|---|---|
| Clinical trial registration | N/A, this study is not an interventional clinical trial. |
| Study protocol | N/A, this study is not an interventional clinical trial. |
| Data collection | Data were collected between 2016-2019 at the Philadelphia VAMC from medical chart review and in-person assessments. A standardized template was used to extract the following variables from the electronic medical record: demographics, psychiatric diagnosis and treatment, offending agent and dose (normalized using chlorpromazine equivalents, where possible), and interfering medications. Motor function was assessed using the Unified Parkinson's Disease Rating Scale Part III and gait data were collected using Opal sensors during the instrumented Timed Up and Go and analyzed with APDM's Mobility LabTM (APDM Inc., Portland, OR, USA). Non-motor symptoms were assessed using the Non-Motor Symptoms Questionnaire and RBD Screening Questionnaire, while olfactory function was assessed using the 40-item University of Pennsylvania Smell Identification Test (UPSIT). UPSIT raw scores were converted to age- and sex-specific percentiles based on normative data. |
| Outcomes | Primary outcome included DAT-SPECT result (negative vs. positive). Secondary outcomes included scores on relevant clinical testing that were compared between those with normal vs. abnormal imaging. |
